# Assessing the impact of a business intelligence program on the employability and well-being of low-income women: a quasi-experimental study protocol

**DOI:** 10.3389/fgwh.2025.1617845

**Published:** 2025-11-13

**Authors:** Marco Faytong-Haro, Alonso Quijano-Ruiz, Daniel Sanchez-Pazmiño, Patricio Alvarez-Muñoz, Jose Diaz-Montenegro, María José Delgado-Rendon, Stephanie Gallegos-Caamaño, Andrea Angulo-Prado, Manuel Murrieta-Vásquez, Karla Robles-Velasco, Ivan Cherrez-Ojeda, Angélica-María Sánchez-Riofrío, Mónica Izurieta Guevara, Claudia Reytor-González, Daniel Simancas-Racines

**Affiliations:** 1 Facultad de Investigación, Universidad Estatal de Milagro, Milagro, Ecuador; 2 Instituto de Investigación, Universidad Agraria del Ecuador, Guayaquil, Ecuador; 3 Research Department, Ecuadorian Development Research Lab, Daule, Ecuador; 4 Facultad de Ciencias Sociales, Educación Comercial y Derecho, Universidad Estatal de Milagro, Ecuador; 5 Universidad Agraria del Ecuador, Guayaquil, Ecuador; 6 Centro de Investigación, Instituto Superior Tecnológico Argos, Guayaquil, Ecuador; 7 Facultad de Administración y Negocios, Universidad Casa Grande, Guayaquil, Ecuador; 8 Facultad de Ciencias de la Salud, Universidad Espíritu Santo, Samborondon, Ecuador; 9 Research Department, Respiralab Research Group, Guayaquil, Ecuador; 10 Facultad de Emprendimiento, Negocios y Economía, Universidad Espíritu Santo, Samborondon, Ecuador; 11 Research Committee, EQ-Lab, Raleigh, NC, United States; 12 Facultad de Ciencias de la Salud Eugenio Espejo, Centro de Salud Pública y Epidemiología Clínica (CISPEC), Universidad UTE, Quito, Ecuador; 13 Escuela de Medicina, Sede Santo Domingo, Pontificia Universidad Católica del Ecuador, Santo Domingo, Ecuador; 14 Facultad de Salud y Bienestar, Pontificia Universidad Católica del Ecuador, Quito, Ecuador

**Keywords:** business intelligence training, employability, gender gap in STEM, data analytics education, quasi-experimental design, economic empowerment, low-income women

## Abstract

Women are underrepresented globally in the field of data analytics, particularly in underdeveloped countries. We present a protocol to assess the impact of the *New Dimensions* program, a data analytics and business intelligence course sequence that aims to address this gender gap by providing free business intelligence training to disadvantaged women in Ecuador. The program offers both technical (Business Intelligence) and soft skills training, including Excel, Power BI, SQL, GitHub, R, Tableau, statistics, Python, and workshops on empowerment, employability, and public speech. The purpose of this quasi-experimental study is to assess the impact of this training program on employability and other well-being outcomes of the participants. A total of 80 individuals will be part in the study, of which 70 will be selected to participate in the program, 50 will receive both hard and soft skills training, and 20 only soft skills training. Ten individuals will form part of the control group with no intervention. The study design involves a nonrandomized control group composed of rejected applicants. Data will be collected through an online application form and a computer-based exam. The outcome measures are participants' labor market outcomes, income, food security, and economic stratification, among others. This protocol will prospectively evaluate the program's potential effectiveness; findings will inform future, larger randomized studies focused on employability and well-being in underrepresented groups.

## 1 Background

The demand for data professionals is high in Ecuador. A recent study found that 94% of business executives in the provinces of Santa Elena, Guayas, Bolívar, Los Ríos, and Galápagos reported needing at least one data scientist in their companies. However, only 20% of science jobs in Ecuador are occupied by women (1, 2), and only 27% of Ecuadorian women (13.1% less than their male counterparts) are employed in the formal sector of the economy (3). This significant gender disparity underscores the urgent need for targeted interventions aimed at improving the employability and career prospects of women, particularly in emerging fields like data analytics and Business Intelligence.

Women in Ecuador are significantly underrepresented in STEM fields, including data science and technology (3, 4). According to the National Institute of Statistics and Censuses (INEC), they make up only 20% of the workforce in science, technology, engineering, and mathematics roles, despite comprising nearly half of the overall labor force. Ecuador also faces a 13.1% gender employment gap in the formal sector, with women from low-income and rural areas experiencing particularly limited opportunities for career advancement (3).

These challenges are compounded by insufficient access to technical and professional development in emerging sectors like data analytics, where demand is rapidly growing (5). Beyond skills deficits, workplace well-being and work–family balance shape participation and retention in STEM employment. Organizational frameworks emphasize attitudinal and behavioral pathways linking engagement, turnover intention, and well-being outcomes (6–8). This is especially concerning given the global rise in demand for data professionals. The U.S. Bureau of Labor Statistics projects a 35.8% increase in data science jobs from 2021–2031, with around 40,500 new openings (9). However, women hold only 18% of these positions, and the gap is even wider in low-and-middle-income countries (LMICs) like Ecuador (5–8). This gender disparity in STEM fields creates significant barriers to economic empowerment and career advancement for women in LMICs (10–12).

Given these global and local challenges, it is clear that targeted interventions are needed to address the specific barriers faced by women in STEM, particularly in the data industry within LMICs. Although previous studies have shown that education and skills training positively impact women's employability and well-being in STEM fields (13–17), a gap remains in understanding the specific challenges faced by women in the data industry in LMICs. Ecuador, classified as an LMIC by the World Bank, exemplifies the need for such targeted interventions to address the workforce disparities in rapidly growing sectors like data analytics (18). Research has demonstrated that enhancing technical skills such as coding, data analysis, and Business Intelligence can increase employment opportunities and wages for women (19–23). Furthermore, developing soft skills such as employability, empowerment, and public speaking is essential for improving women's success in the workplace (24–28). Programs combining both technical and soft skills training have shown great promise in supporting career advancement and overall success for women in STEM fields (29–34).

Despite these encouraging findings, few studies have examined the effectiveness of such interventions in LMICs' data industries. In addition to employability, the New Dimensions program is expected to positively influence broader well-being outcomes, such as food security, health status, quality of life, and household dietary diversity. Studies have shown that improvements in employment and income can lead to better access to nutritious food and healthcare, which in turn enhances household dietary diversity and overall health status (35–37). By increasing participants' economic standing through employment, the program is expected to reduce food insecurity and improve quality of life, contributing to greater household stability and well-being (38). These secondary benefits are particularly important in low-income populations, where employment gains can have a significant impact on both individual and family well-being (39).

Within the Ecuadorian context, soft skills development is recognized as a critical factor for enhancing women's participation and empowerment in the labor market (40). However, there are limited structured opportunities, especially for women from low-income and rural areas, to acquire essential soft skills such as employability, leadership, and public speaking (41). These skills are necessary for navigating workplace challenges and advancing in male-dominated fields such as data science and technology (30, 31, 42).

To address this gap, the New Dimensions program, developed by the Guayaquil-based NGO Ecuadorian Development Research Lab (LIDE), provides a comprehensive approach that combines technical training in data analytics and Business Intelligence with tailored soft skills workshops. Specifically designed for low-income women in Ecuador, the program equips participants with essential digital and interpersonal skills needed to succeed in the growing data industry. This study aims to evaluate the program's effectiveness not only in reducing gender disparities within Ecuador's data sector but also in enhancing broader well-being outcomes, such as improved employment opportunities, increased income, food security, and overall quality of life for participants and their households.

## 2 Research questions

- Does the New Dimensions program improve the employability of low-income women in Ecuador, as measured by changes in employment status and income?
- How does participation in the program affect broader well-being outcomes, such as food security, health status, and quality of life?
- What are the differential effects between participants who receive both technical and soft skills training vs. those who receive only soft skills training?

## 3 Rationale

This study seeks to evaluate the impact of the New Dimensions program on improving both the employability and broader well-being outcomes of low-income women in Ecuador. The significant underrepresentation of women in the data science and Business Intelligence fields, especially in LMICs like Ecuador, restricts their access to well-paying jobs and career advancement opportunities. Addressing this gap is essential to promote gender equality and support broader economic growth.

Technical skills in data analytics and Business Intelligence are increasingly sought after in the labor market. By offering these skills through training, women can improve their employment prospects and income levels. However, employability is not solely dependent on technical expertise. Soft skills such as empowerment, employability, and public speaking are crucial for navigating the competitive labor market and achieving long-term career success.

Research has identified a clear gap in understanding how these combined technical and soft skills programs affect women in LMICs, particularly in terms of secondary outcomes such as food security, health status, quality of life, and household dietary diversity. The New Dimensions program, which combines both technical training and soft skills development, offers a unique opportunity to assess the relative contributions of each type of training to improving not only employability but also participants' overall well-being. This study will contribute valuable evidence on the effectiveness of such programs in addressing gender disparities and supporting the economic empowerment of women in Ecuador.

## 4 Objectives

### 4.1 Primary objective

To evaluate the impact of the New Dimensions program on the employability of low-income women in Ecuador by assessing changes in employment status, income, and socioeconomic status between participants and a control group.

### 4.2 Secondary objectives

- To examine the effects of the New Dimensions program on secondary outcomes such as food security, health status, quality of life, and household dietary diversity among participants.
- To assess the differences in employment status, income, and well-being outcomes between participants who received the New Dimensions program and those in the control group.
- To assess the sustainability of the program's impacts by evaluating changes in the primary and secondary outcomes one year after program completion.

## 5 Methodology & study compoment

### 5.1 Study design

The goal of this study is to analyze whether the New Dimensions program impacts the employability and well-being of its participants, including aspects such as food security, health, and economic stratification. To assess the effectiveness of the program, we employ a three-armed quasi-experimental panel design with two intervention groups and one control group. Participants will complete questionnaires at three time points: baseline (at the beginning of the program), immediately after the program ends, and one year post-program. The intervention groups will be compared to the control group to evaluate the program's impact over time, allowing for a comprehensive understanding of its effects on employability and well-being outcomes.

The first intervention group will be composed of participants who receive both technical and soft skills training, while the second group will be composed of participants who receive only the soft skills component of the program. Both the groups will receive their corresponding interventions simultaneously.

In addition to these intervention groups, a control group will be established made of individuals who reached the final stage of the selection process but narrowly missed being admitted to the full program. This control group allows for comparisons between those who received interventions and those who did not to better understand the impact of the program on employability and wellbeing outcomes, encompassing food security, health, and economic stratification.

The detailed description of participant selection, control group formation, validated measurement tools, and analytical steps provided in this manuscript ensures transparency, facilitating methodological replication and adaptation to similar contexts.

### 5.2 Eligibility criteria and selection process for new dimensions

Low-income, women-at-risk university students and graduates will be invited through social media, university talks, and other channels to apply for the program. After conducting market research, we found that in the two main Ecuadorian cities, Quito and Guayaquil, the demand for data-intensive roles is greater. As a result, the program will be primarily promoted for women living in either of these two cities.

Participants in the program will be selected after a holistic application process, which will consist of three stages. During the first stage, the candidates have to submit an application form. They will be asked basic personal, academic, and socioeconomic information, as well as two short essay questions in which the applicant have to provide a brief description of their current personal situation and their motivation to apply, respectively. A grader will assign a maximum score of 3 for the “personal situation” short-essay question and a maximum score of 5 for the “motivation” one.

Applications with a “motivation” score of less than or equal to 1 which is considered low will be automatically rejected. Then, all applications with a “motivation” and “personal situation” combined score of less than or equal to 2 (25% out of 8 maximum points) will be rejected as well. All non-rejected applicants at this stage will advance in the application process. The reason behind the decision to reject applicants with low scores is to ensure that only the most motivated candidates are given the opportunity to advance to the next stage.

At the second stage, candidates will be invited to take a computer-based basic mathematical skills exam. Applicants who attend the mathematical skills test will also be invited to the final stage of the process, a 15 min interview. A group of four interviewers will ask applicants six broad questions regarding their socioeconomic status, time management skills, knowledge and interest in data, and the *New Dimensions* program. A rubric will be culturally designed to evaluate all applicants on the clarity of their answers, as well as their motivation.

Taking into consideration exam scores as well as interview scores, we anticipate that 70 candidates will be accepted to the program. 50 will be included in the full intervention group, and 20 candidates will be selected for the soft skills component of the program; they are known as the partial intervention group. An additional 10 candidates will be selected from the pool of applicants who narrowly miss being accepted into the program and will be assigned to the control group, which will have no intervention.

The interview guidelines and rubric will be designed after evaluating the applicant pool to ensure a tailored and contextually appropriate selection process. The rubric will assess multiple criteria, including applicants' clarity and coherence of responses, which reflects their ability to clearly and logically express their thoughts during the interview. It will also evaluate motivation and commitment, particularly their interest in the New Dimensions program and future career goals. Relevance of experience or academic background will be considered to assess how well the applicant's previous experience aligns with the skills taught in the program. Socioeconomic need will be another key criterion, with priority given to those from underprivileged or at-risk communities. Time management skills, especially the ability to balance the program with other commitments, will also be evaluated. Lastly, the rubric will assess the potential for success, offering a holistic evaluation based on overall interview performance. The rubric will remain flexible to allow adjustments depending on the overall strength of the applicant pool; for instance, if the pool is stronger than expected, the criteria will become more stringent to ensure fair and meaningful differentiation between candidates.

The allocation of 50, 20, and 10 participants to the full intervention, partial intervention, and control groups, respectively, considers the program's merit-based selection and its practical constraints. It balances the desire to provide comprehensive training to a larger cohort against the anticipated lower engagement from the partial intervention group, who receive less training, and the control group, who may be less motivated due to not being selected for any training. This structure aims to ensure an effective assessment of the program's impact while navigating the limitations of participant availability and commitment.

### 5.3 Interventions and control groups

**Full intervention group:** Participants in this group will undergo comprehensive training encompassing both technical and soft skills. The technical curriculum covers a range of software programs including Microsoft Excel, Power BI, SQL, GitHub, R, Tableau, statistics, and Python. These technical sessions will be delivered remotely in one-hour seminars, four days a week, with each software program's training lasting between 2 and 5 weeks starting September 2025. In addition to technical training, participants will also engage in soft skills development through workshops focused on empowerment, employability, and public speaking. These workshops will consist of four-hour in-person sessions held on weekends, approximately every three weeks. To culminate their learning experience, participants will be required to complete and present a capstone project.

**Training dose and supports:** Technical modules total approximately 80–120 contact hours across Excel, Power BI, SQL, GitHub, R, Tableau, statistics, and Python (1 h sessions, 4 days/week, 2–5 weeks per module), complemented by weekly office hours and pre-class micro-tasks (15–30 min). Soft-skills workshops (empowerment, employability, public speaking) total ∼24 h (in-person, weekend sessions). The capstone requires ∼30–40 h of independent/team work culminating in a public presentation with rubric-based assessment.

**Partial intervention group:** This group will receive a more focused intervention, limited to workshops on empowerment, employability, and public speaking. This targeted approach allows for the assessment of the specific impact of soft skills training on participant outcomes.

**Control group**: Comprising individuals who just miss the cut for full program admission, this group will not receive any intervention. This setup enables a clear comparison to be made between those who receive interventions and those who do not, providing insight into the program's overall effectiveness. Participants for both the partial intervention and control groups will be selected randomly from a pool of applicants who narrowly miss selection for the full intervention, ensuring no significant differences in observable characteristics among the groups.

We initially considered including a fourth group that would receive only technical skills training to gain more insights into the isolated effects of this type of training. However, this is not feasible due to resource and logistical constraints, as well as the funding requirements from the U.S. Embassy, our main funder for the program's execution. The Embassy emphasized the importance of soft skills development—such as communication, public speaking, and leadership—as critical components for promoting long-term employability and economic empowerment for women. Given these requirements, all participants in the intervention groups were required to receive some level of soft skills training. As a result, we opted for two intervention groups: one combining both technical and soft skills training, and one focused solely on soft skills. The distribution of participants across the intervention groups reflects the goal of ensuring that the majority received the comprehensive treatment. Future research could explore the effects of technical skills training in isolation.

### 5.4 Questionnaires

The following validated questionnaires, widely used in Spanish-speaking populations, will be administered to gather comprehensive data from the participants, assessing various aspects of their demographic background, health, socioeconomic status, food security, and quality of life:

- General Demographic Questionnaire: This questionnaire will gather basic information about the participants, such as age, education, and employment status.
- SF-36: This is a widely used self-reported measure of health status that assesses eight domains of health-related quality of life: physical functioning, role limitations due to physical health, bodily pain, general health perceptions, vitality, social functioning, role limitations due to emotional problems, and mental health (43).
- Survey of Socioeconomic Level Stratification: A questionnaire will be employed to know about the socioeconomic status of the participants based on factors such as income, education, and occupation (44).
- HFIAS (The Household Food Insecurity Access Scale): This is tool used to assess household food insecurity (45). It will provide insight into the participants' access to nutritious and adequate food.
- Latin American-Spanish adaptation of the WHOQOL-100 (World Health Organization Quality of Life Questionnaire: This is a self-reported questionnaire that assesses an individual's subjective perception of their quality of life across four domains: physical, psychological, social, and environmental (46).

Each of the validated questionnaires that will be used in this study is available in detail through their respective references provided in the manuscript. These references include comprehensive information on the structure, application, and validation of each tool.

### 5.5 Baseline, endline, and follow-up assessments

Participants will be asked to complete the questionnaires at three time points: baseline (the month of the start of the program), immediately following the completion of the *New Dimensions* program (endline), and one year after the end of the program (follow-up). This will allow us to track changes in outcomes over time and assess the long-term impact of the program on participants. Participants will receive the questionnaires electronically and be given a set amount of time to complete them. Reminders will be sent to those who have not completed the questionnaire in a timely manner. All information collected will be kept confidential and will be used only for research purposes.

### 5.6 Contingency plan

To mitigate any potential challenges that may arise during the implementation of the program, such as participant dropout, logistical delays, or incomplete data collection, we have developed a contingency plan. If participants drop out, we will recruit from a waitlist of eligible candidates to maintain group sizes. For logistical issues, such as delays in workshops or technical difficulties in remote training, we will offer alternative scheduling or supplemental online materials. Additionally, if data collection is incomplete, we will send reminders and offer flexibility in deadlines for questionnaire submissions. These measures ensure that the study can proceed as planned and still provide robust results.

Retention protocol: We will use a standardized contact cadence (SMS/Whastapp/email at T + 3, T + 7, T + 14 days from survey invitation; phone call at T + 21), modest incentives at endline/follow-up, and alternate contacts captured at baseline. Non-responders will be re-contacted in two additional waves at 2-week intervals.

To effectively minimize research biases, the study incorporates clearly defined eligibility criteria and randomized participant assignment for the partial intervention and control groups, significantly reducing selection bias. We will also conduct propensity-score IPTW sensitivity analyses to address potential baseline imbalances. Measurement bias is controlled by employing validated data-collection instruments and standardized interviewer training, ensuring consistency across assessments. Furthermore, attrition bias will be actively managed through systematic follow-ups, routine participant reminders, and timely replacement of dropouts using the established waitlist.

### 5.7 Outcomes

We will measure several outcomes of interest using the mentioned questionnaires.

#### 5.7.1 Primary outcomes

- Employment status: Participants will be asked to self-report their current employment status as full-time, part-time, or unemployed.
- Monthly income: Participants will be asked to report their monthly income in US dollars (Ecuador's national currency).
- Socioeconomic status: This variable will be assessed using the Survey of Socioeconomic Level Stratification Questionnaire, a validated tool used in Ecuador to measure the socioeconomic status of households (44). Participants will be asked a series of questions related to their household assets, income, and education level.

Objective verification (when consented): At endline and 12-month follow-up we will seek employer confirmation (short phone/email check), accept documentary evidence (e.g., contract or pay-stub upload), and record LinkedIn employment updates. Objective indicators will be used to triangulate self-reports.

#### 5.7.2 Secondary outcomes

- Health status will be assessed using the Short Form 36 (SF-36), which measures physical and mental health status across eight domains: physical functioning, role limitations due to physical health, bodily pain, general health perceptions, vitality, social functioning, role limitations due to emotional problems, and mental health. Scores are scaled from to 0–100, with higher scores indicating a better health status for each domain.
- Quality of life: This variable will be assessed using the Latin American-Spanish adaptation of the World Health Organization Quality of Life-100 (WHOQOL-100). The questionnaire consisted of 100 items that assessed an individual's perception of their quality of life across six domains: physical health, psychological health, social relationships, environment, spirituality, and personal beliefs. Scores are scaled from to 1–5, with higher scores indicating better quality of life.
- Food security status: This variable will be assessed using the Household Food Insecurity Access Scale (HFIAS), which includes nine questions related to food insecurity over the past 30 days. Responses will be scored on a scale of 0–3, with higher scores indicating greater food insecurity. The total score will be used to determine the level of food insecurity experienced by households.
- Household dietary diversity: This variable is assessed using the Household Food Insecurity Access Scale (HFIAS), which includes three questions related to the variety of foods consumed by the household. Responses will be scored on a scale of 0–3, with higher scores indicating greater dietary diversity. The total score will be used to determine the household dietary diversity.

### 5.8 Sample size

The sample size for this study will consist of 80 participants, including 50 in the full intervention group, 20 in the soft skills-only group, and 10 in the control group. This study is exploratory in nature due to resource constraints, particularly funding limitations and logistical challenges in delivering training sessions. The sample size was determined based on the maximum feasible recruitment capacity within the available resources rather than a formal statistical power calculation. While a larger sample would enhance the statistical power of the findings, this study serves as an initial investigation into the effectiveness of the New Dimensions program, providing preliminary evidence that can inform future large-scale studies and programs.

### 5.9 Recruitment

The *New Dimensions* program, which is funded by the United States Embassy and Consulate in Ecuador, will be advertised on the social media platforms of the Ecuadorian Development Research Lab (LIDE) and Embassy/Consulate. Furthermore, webinars shared by university authorities will be held to explain the purpose of the program and the steps to apply. We expect that word-of-mouth advertising will help recruit prospective candidates for the program. Prospective students will be directed to the *New Dimensions* website, which includes a button to access an application form in Qualtrics. It is important to note that while the program is financed by the embassy, the research evaluation component of the program is funded by Instituto Tecnológico Argos.

### 5.10 Assignment of interventions

The full intervention group, comprising both hard and soft skills training, will be selected from the full pool of applicants based on economic need, academic, and motivational merit by a team of four reviewers. Thus, owing to the nature of this application process, the intervention for the full group will not be randomly assigned. A pool of applicants who narrowly misses being selected for the full intervention group will be created. Participants from the soft skills-only (partial intervention) and control groups will be randomly selected using Microsoft Excel's random-number generator function. Random selection will be conducted within strata defined by city (Quito/Guayaquil), baseline employment status (employed vs. not), and motivation score tertiles to minimize imbalance. Excel RAND() will be used to generate random numbers separately within each stratum, followed by rank-ordering and assignment.

## 6 Data collection, management, analysis and ethics

### 6.1 Data collection methods

Candidates will submit an online application form in which they have to provide demographic and socioeconomic information as well as their motivation to apply to the program. After submitting this form, the shortlisted candidates will be asked to take a computer-based exam to collect information on their math skills. Applicants will be encouraged to answer all questions. Each candidate who advances to the next stage of the selection process will be interviewed by members of the program's admission committee. All interviewers will record and score their responses. At follow-ups, we will implement the objective employment verification procedures (employer check, optional document upload, LinkedIn observation) for consenting participants.

### 6.2 Data management

To ensure the security and confidentiality of the data collected from the study, all information regarding the candidates and their scores will be managed and stored on a secure cloud-based platform. Access to the data will be restricted to the research team and authorized personnel, and appropriate measures will be taken to prevent unauthorized access to or disclosure of sensitive information. Data backups will be created regularly to prevent loss of or damage to the data, and all necessary precautions will be taken to ensure the integrity and reliability of the data throughout the study.

### 6.3 Statistical plan

Descriptive statistics will be used to summarize the demographic characteristics of the study population and examine the distribution of outcome variables. Continuous variables will be summarized as means and standard deviations or medians and interquartile ranges depending on their distribution. Categorical variables will be summarized as frequencies and percentages.

To assess the effectiveness of the *New Dimensions program*, we employ a quasi-experimental design with two intervention groups and one control group. The two intervention groups will be compared to the control group to evaluate the impact of the program on employability and well-being outcomes.

To test for differences in baseline characteristics between the intervention and control groups, we used *t*-tests for continuous variables and chi-square tests for categorical variables. To examine changes in the outcome variables over time, we plan to employ linear mixed-effects regression models. Models will include fixed effects for group (full/partial/control), time (baseline/endline/12-month), and the group × time interaction (primary estimand), with random intercepts for participants. These models will allow us to account for the within-subject correlation of repeated measurements over time and adjust for potential confounders, such as age and educational level. We will additionally adjust for city, baseline employment status, and motivation score (tertiles). This will help to control for variations in motivation that could impact outcomes, thereby reducing the risk of false positives. By adjusting for motivation, we aim to ensure that the observed effects are more likely to be attributable to the intervention itself rather than pre-existing differences between groups.

Missing data will be handled using complete case analysis when the proportion of missing data is low and assumed to be missing completely at random (MCAR). For cases where missingness is more substantial, we will implement inverse probability weighting (IPW) to adjust for potential differences between participants who remain in the study and those lost to follow-up. Descriptive comparisons between retained and lost participants will also be performed to assess systematic differences that could influence the results.

All statistical tests will be two-sided, with a significance threshold of *p* < 0.05. Missing data will be addressed using Multiple Imputation by Chained Equations (MICE) under MAR assumptions, with sensitivity analyses including complete-case and IPW approaches. Given the exploratory sample size (*n* = 80), we will emphasize precision of estimates (95% confidence intervals) rather than sole reliance on *p*-values, and will apply small-sample robust standard errors where appropriate. All analyses will be conducted in Stata 19.0.

To prevent undisclosed flexibility in our analysis pipeline, as mentioned before, we have predefined all primary and secondary outcomes and will adhere strictly to the outlined statistical methods. Outcome-neutral conditions are established by employing validated instruments known to avoid floor or ceiling effects. Given that the study is exploratory due to funding and resource constraints, no formal power calculation was performed. Therefore, some analyses, particularly those involving subgroup comparisons (e.g., differences between full and partial intervention groups), should be considered exploratory. Assumptions for our analyses include approximate normality of continuous variables and that data are missing at random (MAR), both of which will be examined through diagnostic and sensitivity checks. Missing data will be addressed using Multiple Imputation by Chained Equations (MICE) under MAR assumptions, and effect sizes with 95% confidence intervals will be reported and compared across imputed and complete-case approaches. Robustness will be evaluated by repeating models both with and without adjustment for baseline motivation scores. Finally, to address potential selection bias from the non-random assignment of participants to the full-intervention group, we will perform propensity-score inverse probability of treatment weighting (IPTW) sensitivity analyses based on age, education, city, baseline employment status, and motivation score.

### 6.4 Ethics

This research project was approved by the Institutional Review Board (IRB) of Kennedy Clinic in Guayaquil, with approval code HCK-CEISH-2022-006. Written informed consent will be obtained and will be required at every stage of the research in accordance with ethical guidelines for human subjects' research.

### 6.5 Limitations

One limitation of this study is the relatively small sample size, which may affect the generalizability of the findings. While this limits the extent to which broad conclusions can be drawn, the study serves as an important first step in evaluating the impact of the New Dimensions program. The results can provide preliminary insights and a foundation for future, larger-scale studies that can offer more concrete recommendations for policymakers, educators, and professionals in shaping educational interventions. Additionally, this study aims to contribute to the broader understanding of the impact that programs like these can make in addressing gender disparities in the labor market, particularly in STEM fields. By providing initial evidence, this research can inform the development of similar programs and further our knowledge of how targeted educational interventions can improve employability and well-being outcomes for women, especially in low-and-middle-income countries.

Another limitation of the study design is the absence of a “technical training only” group, which prevents us from isolating the specific effects of technical training apart from the soft-skills component. This decision was driven by resource constraints and funders' requirements, which prioritized soft-skills development as essential for long-term employability and well-being. As a result, all intervention participants received some level of soft-skills training. While this limits our ability to evaluate the standalone impact of technical training, the study still provides valuable insights into the combined effects of technical and soft-skills training on employability and well-being. Future research could incorporate a fourth arm consisting solely of technical training to disentangle the distinct contributions of each component. Despite this limitation, the present study makes a contextual contribution by testing a combined technical + soft-skills model for low-income women in an LMIC setting, using validated multi-domain outcomes and objective employment verification.

Additionally, the reliance on self-reported data may introduce bias due to social desirability or inaccurate recall by participants. Although validated questionnaires are used, self-reported measures may not fully capture changes in employability, well-being, or economic status. Additionally, the study's focus on a single geographic region may limit the applicability of the results to other regions or countries, particularly those with different labor market conditions or cultural contexts.

Finally, the study's short-term follow-up period may not capture the full extent of the program's long-term impact on participants. While we assess outcomes one year after the program's conclusion, longer-term tracking would be beneficial to fully understand how the skills acquired affect sustained employability and career progression. We also recommend a scaled-up, fully randomized trial with extended follow-up to robustly isolate causal effects.
